# Cancer Vaccines and Immunotherapy for Tumor Prevention and Treatment

**DOI:** 10.3390/vaccines9111298

**Published:** 2021-11-09

**Authors:** Jagmohan Singh, Wilbur B. Bowne, Adam E. Snook

**Affiliations:** 1 Department of Pharmacology & Experimental Therapeutics, Thomas Jefferson University, 1020 Locust Street, Philadelphia, PA 19107, USA; jagmohan.singh@jefferson.edu; 2 Sidney Kimmel Cancer Center, Department of Surgery, Thomas Jefferson University, 1100 Walnut Street, Philadelphia, PA 19107, USA; Wilbur.Bowne@Jefferson.edu; 3 Sidney Kimmel Cancer Center, Departments of Pharmacology & Experimental Therapeutics and Microbiology & Immunology, Thomas Jefferson University, 1020 Locust Street, Philadelphia, PA 19107, USA

**Keywords:** cancer vaccine, cancer immunotherapy, immune checkpoint blockade, CAR-T cell therapy

## Abstract

In this editorial, we highlight articles published in this Special Issue of *Vaccines* on “Cancer Vaccines and Immunotherapy for Tumor Prevention and Treatment”, recent developments in the field of cancer vaccines, and the potential for immunotherapeutic combinations in cancer care. This issue covers important developments and progress being made in the cancer vaccine field and possible future directions for exploring new technologies to produce optimal immune responses against cancer and expand the arena of prophylactic and therapeutic cancer vaccines for the treatment of this deadly disease.

## 1. Introduction

Cancer immunotherapy comprises a number of current and promising therapeutic approaches intended to eradicate tumors by activating host antitumor immunity [1,2]. FDA approval of immune checkpoint blocking (ICBs) therapies and chimeric antigen receptor (CAR)-engineered T-cell immunotherapies has significantly improved cancer treatment, but they are not effective in all cancer patients. Cancer vaccines have not yet made the same clinical impact but have preventive and therapeutic potential, alone or in combination [3], and may provide lifelong immunity against cancer recurrence, potentially becoming an integral part of future combinatorial immunotherapies [4].

To date, most cancer vaccines have failed to generate optimal immune responses to completely eradicate cancers due to continuous evolution of the tumor microenvironment (TME), imperfect understanding of vaccine components that are ideal to generate robust immune responses against self- or neo-antigens, and/or examination in challenging clinical settings (last line in heavily pre-treated patients). A greater understanding of the TME has helped overcome some of these hurdles to generate successful immune responses against tumor-associated antigens (TAAs) [4]. Moreover, new vaccine platforms and applications in secondary prevention settings [5], continue to shape the path towards making effective cancer vaccines a possible reality. 

## 2. Current Challenges in Developing Cancer Vaccines

Numerous challenges make cancer vaccination difficult, and two review articles in this Special Issue give insight into numerous immunological and technical challenges [4,6]. Development of prophylactic and therapeutic vaccines for cancer is far more challenging than developing vaccines against viral or bacterial diseases because of the limited antigenicity of cancer cells; immune evasion pathways that block T-cell activation, suppress the TME, and others; differences in ideal effector mechanisms to resist microbial pathogenesis (often antibodies) and eliminate cancer (often cytotoxic CD8^+^ T-cells); and many more. Indeed, Sliker and Campbell discuss challenges created by the continuously evolving TME and downstream impacts on the tumor immune landscape [7]. They highlight research showing that fibroblasts in tumors can impact the TME and profoundly affect the generation of immune responses against tumor cells by cell-cell contacts and secretion of various cytokines. Often, the TME can dampen immune responses via immune checkpoint upregulation, limiting intratumoral T-cell function. They suggest that continuous research effort in this area will give insightful information about mechanisms of immune invasion by tumor cells to overcome these barriers. In that context, Chai et al. demonstrate that localized delivery of ICBs in the liver could inhibit hepatic colon cancer metastases by reshaping the TME [8], an approach that might be extended to other clinical settings.

## 3. Tumor Antigens for Cancer Vaccines

Several review articles in this Special Issue emphasized how our understanding of antitumor immune responses has accelerated the identification of tumor antigens which may be recognized by endogenous antitumor immune responses and exploited as vaccine targets for cancer immunotherapy [4,6,9]. These review articles discuss several TAAs, including overexpressed antigens and differentiation antigens. Overexpressed antigens such as mucin 1 (MUC1), HER2/neu, telomerase, and survivin are generally expressed at very low levels in normal cells, but their expression increases in tumor cells, producing an opportunity for targeting by vaccines. Normal differentiation antigens such as gp100 and prostatic acid phosphatase (PAP) are proteins that are normally expressed in differentiated tissues (melanocytes and prostate, respectively) and retained in cancers derived from those tissues (melanoma and prostate cancer, respectively) [6]. Although discoveries in genomics and proteomics have significantly accelerated the identification of tumor antigens in patients, identification of shared tumor antigens remains a challenge for many cancer types, leading to a need for identifying patient-specific tumor neoantigens, which may be a better approach to eradicate tumors by targeting immunogenic and/or driver mutations.

## 4. Success of Preventative Vaccines against Virus-Associated Cancers

While vaccine strategies for cancer are inherently more challenging than those against viruses, cancers of viral origin [hepatitis B virus (HBV) and human papillomavirus (HPV)] provide an excellent opportunity for primary cancer prevention through viral-directed vaccines [6,10]. HBV and HPV infection, major viral causes of hepatocellular and cervical cancer, respectively, have been successfully prevented through FDA-approved vaccines [9]. Vaccines can potentially provide lifelong immunity against infection and can help reduce the significant global burden of these cancers [9]. Complementary to these observations is a study by Ahmed et al. showing that the Epstein–Barr virus (EBV) protein BZLF1 may be a good candidate for dendritic cell (DC)-based vaccines in immunosuppressed solid organ transplants recipients with post-transplant lymphoproliferative disease (PTLD) [10]. Despite the success in preventing viral infections through vaccination, there is limited success in developing therapeutic vaccines targeting viral antigens for the treatment of cancers of viral origin. They suggest that more research is required to understand the causal mechanisms of these cancers, which will help design therapeutic vaccines for the treatment of these cancers at their various stages of development. While success has been made in preventing cancers derived from HBV and HPV, population health approaches are required to increase vaccine uptake in the US and globally [11], and vaccine strategies for other cancer-causing infections remain challenging [helicobacter pylori, hepatitis C virus (HCV), EBV, etc.].

## 5. Recent Developments in Prophylactic and Therapeutic Cancer Vaccines

Review articles in this issue reminded us how the recent SARS-CoV-2 pandemic has boosted awareness of vaccines to prevent communicable diseases and brought a renewed focus on cancer vaccines and improved vaccine technologies [4,5,6]. Renewed interest in vaccine technologies has accelerated scientific interest in using these technologies to design vaccines for cancer immunotherapy. Though novel vaccines developed in 2020 have been a remarkable success for SARS-CoV-2, it is not yet known how these technologies will translate to cancer immunotherapy.

Cancer recurrence after surgery, chemotherapy, radiotherapy, and/or targeted therapies is a major limitation of our current cancer care paradigms. Prophylactic and therapeutic cancer vaccines can help prevent or eradicate metastatic tumors, respectively, significantly reducing the social, emotional, and economic burden of cancer recurrence [6]. To achieve this goal, several approaches are being tested at various stages of pre-clinical and clinical research. To generate a successful immune response against tumor antigens, various methods are being tested to deliver antigens at the tumor site or systemically to elicit tumor-specific T-cell responses. An enduring challenge has been identifying ideal “platforms” to generate robust immune responses based on bacterial or viral or component vaccines containing peptides, RNA, or DNA molecules [4]. Vaccine platforms must overcome hurdles like immune tolerance and pre-existing immunity against specific vectors [4]. Various novel delivery approaches such as lipid nanoparticles (LNPs) and electroporation are being tested in different clinical trials. Research in these delivery approaches may soon result in robust delivery systems to generate tumor-directed T-cell responses and provide benefit to patients. Various delivery approaches may follow different routes of antigen delivery depending on the treatment needs. Electroporation techniques such as electrochemotherapy (ECT) and gene electrotransfer (GET) can deliver antigens directly into tumors or tumor sites to generate robust immune responses [12]. Similarly, regional delivery of ICBs has shown promising results in animal models of metastatic disease [8].

## 6. Adoptive Immunotherapy

Overcoming self-tolerance is a big challenge for vaccines targeting normal antigens. For those reasons’ mechanisms of central and peripheral tolerance and their impact on antitumor immunity require interrogation. Moreover, conventional cancer therapies can have a devastating impact on the patient’s ability to generate successful immune responses against normal or mutated antigens. In contrast, adoptive immune therapies are a passive form of immunotherapy that overcomes many tolerance and patient immune function challenges. In that context, bispecific antibodies (BsAbs) are a novel immunotherapy tool that is not restricted by immune tolerance at it redirects endogenous T-cells of irrelevant specificity to target tumor antigens, and several are being tested in various clinical trials [13]. You et al. [13] has given a detailed overview of BsAbs and their design against tumor antigens and other targets and their usefulness in cancer treatment. BsAbs perform multiple functions by binding to two or more molecules of interest at a time. BsAbs can be useful in blocking oncogenic receptor tyrosine kinases (RTKs) such as EGFR and HER2, blocking angiogenic receptors, and inducing tumor cell death by targeting “don’t eat me” pathways such as the CD47/SIRPα pathway or activating TCR signaling in bystander T-cells. An example of this is FS120 BsAb, which can bind both 4-1BB and OX40 on CD4^+^ and CD8^+^ T-cells to augment their activity. BsAb antibodies can also redirect T cells and NK cells towards tumor antigens to eradicate tumors.

Similarly, Wang et al. identified a novel form of adoptive cell therapy for the treatment of T-cell malignancies [14]. They developed a flexible approach for targeting specific Vβ families of TCRs expressed by T-cell malignancies by combining (1) T cells engineered to express a chimeric CD64 protein that acts as a high-affinity immune receptor (IR) and (2) a monoclonal antibody directed toward a specific TCR Vβ family. This combination approach produced robust in vitro and in vivo efficacy. This proof-of-concept may be translated to patients to produce precise targeting of T-cell malignancies without substantial immune compromise—typical approaches directed at T-cell malignancies may also eliminate the normal T-cell compartment.

## 7. Vaccine Combinations with Other Immunotherapies

Cancer vaccine efficacy might be significantly improved by applying cancer vaccines in combination with other immunotherapeutic approaches, such as ICBs [15]. Over their first decade of clinical use, ICB antibodies have significantly matured as cancer treatment options, though none are entirely successful [15]. Cancer recurrence and metastasis is still a significant challenge to overcome in cancer treatment, despite the deployment of ICBs. Combining ICB antibodies nivolumab (anti-PD-1) and ipilimumab (anti-CTLA-4) is FDA-approved for mismatch repair-deficient/microsatellite instability-high metastatic colorectal cancer reflecting greater efficacy than either monotherapy—though immune-related adverse events (irAEs) are also significantly increased by the combination. In that context, a combination of vaccines with ICBs may increase ICB efficacy against ICB-resistant “cold” tumors with less risk of toxicity [4].

Various clinical trials are testing different cancer vaccines in combination with ICBs, such as OTSGC-A24 peptide vaccine with nivolumab and ipilimumab (NCT03784040) in gastric cancer and the mRNA vaccine, BI 1361849, with durvalumab (anti-PD-1) and tremelimumab (anti-CTLA-4) in NSCLC (NCT03164772). Significant work is needed to achieve maximum benefit with acceptable toxicities, but these initial explorations are the first step on that path. Prime boost immunization strategies with different types of vaccines such as RNA, DNA, and viral/bacterial vector vaccines may enhance efficacy. Indeed, NCT03532217 is exploring a combination of neoantigen DNA vaccine with ipilimumab/nivolumab and the prostate-specific antigen (PSA)-directed vaccine PROSTVAC. PROSTVAC is a diversified prime/boost vaccine containing transgenes for PSA and “TRICOM”, the three T-cell costimulatory molecules B7-1, ICAM-1, and LFA-3 [15].

Chiu et al. reviews immunotherapy and vaccine strategies for surgically-resectable non-small cell lung cancer (NSCLC) [16]. A combination of ICBs with vaccines against various antigens such as EGF, MAGE-A3, and hTERT may provide a therapeutic option to prevent recurrence in NSCLC patients. Importantly, vaccines may be beneficial and may be an alternative choice for NSCLC patients with irAEs from neoadjuvant therapy or at high risk of irAE with other immune therapies. However, there is a critical need for proper molecular diagnostic tests to select patients for combinatorial therapy with ICBs and vaccine therapies [16]. Larger data sets from clinical trials are required for correlational studies of mutational signatures of tumors with ICB + vaccine combinations.

## 8. Summary and Conclusions

Cancer immunotherapy has given cancer patients new hope. While not yet broadly successful, cancer vaccines may be a very important tool in the immunotherapy tool kit for oncologists. The recent SARS-CoV-2 pandemic has renewed interest in vaccine technology which may have downstream impacts on cancer vaccine development. Vaccines are an ideal tool to prevent pathogen-related diseases prophylactically, but challenges remain for creating therapeutic vaccines for established tumors. Since other arms of immunotherapy, such as ICBs and adoptive T-cell therapies, are not completely effective in all the patients, combining them with cancer vaccines may be an area of rapid growth and advancement to treat or prevent metastatic disease [15]. Prophylactic, therapeutic, and personalized cancer vaccines have significant promise, but significant work remains to realize that potential.
